# Exploring vitamin K2 and B12 as bioactive micronutrients for dentin conditioning in regenerative endodontics procedures

**DOI:** 10.1007/s00210-026-05129-8

**Published:** 2026-03-17

**Authors:** Hend Mohamed Kandil, Shereen N. Raafat, Shaimaa M. Gomaa, Shehabeldin Saber, Nawar Naguib Nawar

**Affiliations:** 1 Department of Endodontics, Faculty of Dentistry, The British University in Egypt (BUE), El Sherouk City, Egypt; 2 Department of Pharmacology, Faculty of Dentistry, The British University in Egypt (BUE), El Sherouk City, Egypt; 3 Dental Science Research Group, Health Research Centre of Excellence, The British University in Egypt (BUE), El Sherouk City, Egypt

**Keywords:** Vitamins, Periodontal ligament stem cells, Regenerative endodontics, Osteo/odontogenic differentiation

## Abstract

This study assessed the potential of vitamins K2 and B12 as dentin surface conditioners to modulate the regenerative behavior of human periodontal ligament stem cells (hPDLSCs). hPDLSCs were isolated and characterized by flow cytometry, which confirmed the mesenchymal stem cell phenotype. Cell viability assays showed no cytotoxicity at the tested concentrations; therefore, 16 µg/mL was selected for further analysis. Both vitamins significantly enhanced cell migration, attachment to dentin disks, and mineralization potential, as demonstrated by Alizarin Red staining and alkaline phosphatase activity after 14 days of osteogenic induction. Gene expression analysis revealed that vitamin B12 primarily upregulated RUNX2, whereas vitamin K2 increased the expression of osteoprotegerin, osteocalcin, dentin sialophosphoprotein, and cementum protein 1. Furthermore, both vitamins reduced the expression of nuclear factor kappa-B and interleukin-6 and increased glutathione levels, with vitamin B12 exhibiting stronger anti-inflammatory and antioxidant effects. Despite these variations, no significant differences were observed between the two vitamins in terms of cell proliferation, mineralized nodule formation, or alkaline phosphatase activity, suggesting a comparable overall regenerative potential. These findings indicate that vitamins K2 and B12 are biocompatible and promote favorable hPDLSC responses through distinct molecular pathways. They may serve as practical, chairside-applied micronutrient conditioners in regenerative endodontic procedures, potentially improving the treatment outcomes.

## Introduction

Regenerative endodontic procedures (REPs) are biologically based approaches that aim to restore damaged tissues in immature necrotic teeth (Saber 2009). According to the American Association of Endodontists, successful REP outcomes are assessed using three key criteria: elimination of clinical symptoms and radiographic signs of healing, increased root length and dentinal wall thickness (especially critical in immature teeth), and positive pulp sensibility responses indicating potential regeneration of functional pulp tissue (Galler et al. 2016a). Regardless of whether true regeneration occurs and to what extent (Rosa et al. 2022), there is a consensus that the process is stem cell-mediated, and that its success is contingent upon the effective disinfection of the root canal and successful recruitment of such cells into the root canal space, which in turn can be facilitated by the release of chemotactic endogenous signals from dentin (Atesci et al 2020a; Lovelace et al 2011; Hargreaves et al. 2014).

It is well acknowledged that the dentin matrix serves as a reservoir for different growth factors (Hargreaves et al. 2014). Many studies have shown that the proliferation and differentiation of stem cells can be enhanced by dentin treatments that release trapped endogenous growth factors (Atesci et al. 2020; Ferreira et al. 2020; Zeng et al. 2016). Current guidelines recommend the use of EDTA in the final wash for this purpose (AAE, 2018). While these endogenous growth factors are readily available, stem cell activity can be upregulated or downregulated by a broader set of physical and chemical cues (Alves Berti Pereira et al. 2025; Galler et al. 2016b). Therefore, dentin conditioning is a promising research area that deserves further exploration for novel nonconventional biomodulators, such as vitamins.

The literature indicates that certain vitamins, such as B12 and K, have favorable regenerative effects (Wheeler et al. 2020). Therefore, incorporating them to condition root canals during REP protocols has the potential to improve treatment outcomes. Empirical evidence has shown that vitamin K, a fat-soluble vitamin, plays a crucial role in the processes of hemostasis and osseous remodeling in vertebrate organisms (Orlando et al. 2015; Vermeer and Braam 2001). Subsequent investigations have underscored the pivotal importance of vitamin K in mitigating the accumulation of prostaglandin E2, which is associated with apoptotic osteoblast cells and is essential for the synthesis of specific coagulation proteins, thereby fostering mineralization via gamma-carboxylation of osteocalcin (Gundberg et al. 2012; Lacombe & Ferron 2015; Muszyńska et al 2020). Also, in vitro analyses have demonstrated that vitamin K possesses the capacity to promote the osteoblastic differentiation of dental pulp stem cells (DPSCs) (Cui et al. 2021).

On the other hand, vitamin B12, commonly referred to as cobalamin, functions as a crucial cofactor for enzymes engaged in DNA synthesis, erythropoiesis, and myelin sheath formation (Neiva et al. 2005; Remacha et al. 1997). As a hydrophilic vitamin, vitamin B12 has emerged as an effective agent for facilitating the regeneration of dental and osseous tissues through the proliferation of DPSCs in controlled laboratory environments (Inamoto et al. 2023).

In summary, both vitamin K2 and vitamin B12 exhibit encouraging effects with possible prospective advantages for their integration into REPs. This study aimed to evaluate the impact of dentine conditioning with either vitamin K or vitamin B12 versus EDTA on the viability, attachment, differentiation potential and oxidative stress of human periodontal ligament stem cells (hPDLSCs). The null hypothesis was that there would be no differences between the compared groups.

## Materials and methods

The Research Ethics Committee of the Faculty of Dentistry at The British University in Egypt granted approval for the experimental design and methodology (FD BUE REC 23–040).

### Materials preparation

Vitamin K₂ (menaquinone-4) was obtained from Sigma-Aldrich (# V9378). A stock solution was prepared with a concentration of 500 mg/L (0.5 mg/mL) in dimethyl sulfoxide (DMSO). Vitamin B₁₂ (Cyanocobalamin) was purchased from Sigma-Aldrich (# V2876). A stock solution was prepared at a concentration of 1 mg/mL in sterile deionized water. The stock solutions were stored in amber glass vials at − 20 °C and renewed weekly to ensure stability per the manufacturer instructions. Working solutions were freshly prepared prior to each experiment by appropriate dilution of the stock solution using the cell culture medium.

### Isolation and culture of hPDLSCs

Impacted mandibular molars (*n* = 3) were procured from the Maxillofacial Surgery Department within the Faculty of Dentistry at The British University in Egypt. After extraction, the teeth were maintained in DMEM/F12 (Dulbecco’s Modified Eagle Medium/F12 Ham medium (#2,715,699, Gibco BRL, CA, USA) supplemented with antibiotics (300 U/mL penicillin and 300 mg/mL streptomycin; Sigma-Aldrich). Stem cell isolation was conducted as previously described (Rady et al. 2024; Shamel et al. 2024). In summary, the periodontal ligaments underwent fragmentation, followed by enzymatic digestion for a duration of one hour at 37 °C. The digestion process incorporated a culture medium containing collagenase I (3 mg/mL) and dispase II (4 mg/mL). DMEM/F12 supplemented with 10% fetal bovine serum (Gibco BRL, CA, USA) and 1% antibiotic/antimycotic (Gibco) was used as the culture medium. Following the preparation, the cells were incubated at 37 °C with 5% CO_2_ in a humidified environment. Regular observation of cells was conducted using an inverted microscope (TCM 400; Labomed, Los Angeles, CA, USA). This investigation employed cells at passage four (P4), ensuring a minimum of three replicates for each experimental group.

### Characterization of the isolated hPDLSCs

The expression of specific surface antigens was determined by flow cytometry as previously described (Aqabat et al. 2024). hPDLSCs were collected at passage 4 and fixed with 4% paraformaldehyde for 15 min. The cells were then incubated with a 3% solution of bovine serum albumin, followed by exposure to primary antibodies that specifically targeted CD45, CD34, CD90, CD73, and CD105 for 1 h. Following incubation, the cells were thoroughly rinsed with wash buffer, and secondary antibodies (BD Biosciences, Piscataway, NJ, USA) were applied for 45 min at ambient temperature. Subsequently, the cells underwent three washing cycles and were then analyzed using a flow cytometer (Elashiry et al. 2023).

Multilineage differentiation was performed using a commercially accessible “Human Mesenchymal Stem Cell Functional Identification Kit” (#SC006; R&D Systems Inc.) (Saber et al. 2023a). This kit comprises specialized media supplements tailored for the processes of osteogenesis, adipogenesis, and chondrogenesis. These supplements were meticulously formulated to promote cell differentiation into osteogenic, adipogenic, and chondrogenic lineages. The cells were cultured in a 24-well plate with specific media corresponding to each differentiation type (osteogenic, adipogenic, and chondrogenic) over a period of three weeks Osteogenesis was evaluated using Alizarin red staining (# SHBL6801, Sigma-Aldrich, St. Louis, MO, USA). Adipogenesis was assessed by Oil Red O staining (#A12989.14, Thermo Scientific). Chondrogenic differentiation was confirmed by Alcian Blue staining (#Q05H016, Alfa Aesar, Thermo Fisher Scientific). Finally, the wells were examined using an inverted microscope and images were captured using a digital camera (Heidar et al. 2025).

### Cell viability assessment using MTT assay

The cytotoxic effects of vitamins K2, B12, and EDTA were evaluated using a 3-(4,5-dimethylthiazol-2-yl)−2,5-diphenyltetrazolium bromide (MTT) assay (Elashiry et al. 2023; Zand et al. 2023). Serial dilutions of EDTA and both the vitamins were prepared at a starting concentration of 16 µg/mL. Human periodontal ligament stem cells (hPDLSCs) were cultured (1 × 10^4^ cells/well) for 24 h in 96-well plates with 180 μL DMEM. Following the introduction of the material extract, the cells were cultured for 1, 3, and 7 d at 37 °C in an atmosphere of 5% CO_2_. At designated intervals, the samples were incubated for 4 h after treatment with 1 mg/mL MTT solution. Subsequently, each well received 0.2 mL of dimethyl sulfoxide (DMSO) was added to each well to dissolve the formazan crystals generated by living cells via MTT reduction. A spectrophotometer was used to measure the violet color produced (570 nm wavelength) (Aljabri et al. 2025). The experiment was conducted thrice, and all samples were analyzed in six replicates (*n* = 6). The data collected were normalized using cells in a normal medium using the following equation:

$$\%\;Viability\;=\frac{Absorbance_{test}-\;Absorbance_{blank}}{Absorbance_{Control}-\;Absorbance_{blank}}\;\times\;100$$

### Scratch wound assay

Cell migration and proliferation were assessed using a scratch wound healing assay (Sayed et al. 2023). hPDLSCs were cultured (30 × 10^4^ cells/well) in sterile 6-well plates until they occupied 95–100% of the plate surface. Cells without vitamins in the medium were used as negative control. A sterile pipette tip was used to make a scratch in each sample, and the culture medium supplemented with vitamins was then applied to the hPDLSCs. A phase-contrast inverted microscope was used to view the induced wounds in each well at 0, 24, 48, and 72 h. The wound area was evaluated using ImageJ software (Bethesda, MD, USA) and the percentage of scratch closure was calculated. Each group was assessed in triplicate and the assay was run three times.

### Scanning electronic microscopy (SEM)

To shed light on the attachment of cells to dentin disks after culturing with both vitamins, SEM imaging was performed. Nine dentin disks (*n* = 3 per group) were grown separately in 24-well plates. hPDLSCs (10 × 10^3^ cells/well) were added to dentin disks and cultured for 72 h. The cells were then fixed by subjecting them to a 4% glutaraldehyde solution in PBS for 4 h at 4 °C. All dentin disks were dehydrated using a series of increasing ethanol concentrations. The samples were then subjected to gold sputtering using a sputter-coating process (15 mA for 4 min) (HUMMER 8.0, ANATECH). The disks were analyzed using scanning electron microscopy (SEM, Leo Supra 55) (Saber et al. 2023a).

### Osteogenic differentiation of hPDLSCs

For osteogenic induction, an induction medium was employed, which included α-MEM, 10% FBS, dexamethasone 0.1 µM (Sigma), beta-glycerophosphate 10 mM (Merck, Darmstadt, Germany), and ascorbic acid-2-phosphate (200 µM) (#50–81-7, TOCRIS, Bioscience, UK). The cells were cultured in 6-well plates until they reached 70% confluence. The normal culture medium was then replaced with osteogenic medium conditioned with Vit B12 or Vit K2. Osteogenic induction was performed for 14 days and the medium was changed twice per week. Cells in regular culture medium constituted the negative control group, whereas those in osteogenic medium devoid of vitamins were used as the positive control group (Rady et al. 2024; Hafez et al. 2024).

#### Cell mineralization assay

The degree of mineralization was measured by Alizarin Red staining (Saber et al. 2023a). The osteogenic medium was carefully removed and the cells were fixed for 15 min with 10% formaldehyde. The monolayers were rinsed twice with distilled water, and then Alizarin Red stain solution (pH 4.1) was added. The plates were kept at room temperature for half an hour with continuous gentle shaking. The red-stained mineralized nodules were imaged using an inverted microscope and then solubilized with a 10% warm acetic acid solution. Aliquots from each well were transferred to a 96-well plate and the color was quantified by spectrophotometry at 405 nm.

#### Alkaline phosphatase (ALP) enzyme activity determination

The activity of the ALP enzyme was determined by studying the kinetics of the conversion of colorless para-nitrophenylphosphate (p-NPP) to a yellow para-nitrophenolate (p-NP) (Aqabat et al. 2024) by the action of the enzyme. Briefly, the monolayers were rinsed with water and once again with room-temperature alkaline phosphatase buffer (ALPB). The monolayers on a 6-well plate were treated with 1 mL of ALPB, and each well was filled with an equal volume of p-NPP reagent (#XG3546131, Thermo Scientific) to produce a yellow solution owing to the formation of yellow p-NP. In a 96-well plate, 50 μL of the yellow medium was immediately mixed with an equivalent volume of NaOH to halt the reaction. In the previous step, the procedure was repeated every minute for 10 min for each group. The yellow color was measured using spectrophotometry at a wavelength of 405 nm. The rate of p-NP accumulation over time was plotted, and the slope of each reaction (group) was calculated to determine the pace of the reaction reflecting enzyme activity (El Shafei. et al., 2023).

#### Osteo/odonto/cementogenic markers measurement

The expression of osteo/odontogenic and cementogenic markers was assessed using quantitative real-time polymerase chain reaction (qRT-PCR) (Table 1). Following the manufacturer’s instructions, cells were collected, and total RNA was extracted using the RNeasy Mini Kit (#160,048,808, QIAGEN). A nanodrop spectrophotometer (Thermo Fisher, USA) was utilized to ascertain the extracted RNA concentration and purity. The RNA samples were retrotranscribed using a RevertAid First StrandcDNA Synthesis Kit (#01266358, Thermo Scientific) and amplified using Maxima SYBR Green qPCR Master Mix (#2,867,667, Thermo Scientific). The PCR thermal cycling parameters were as follows: 2 min at 50 °C, 30 s at 95 °C, 35 cycles at 95 °C for 10 s, and 40 s at 60 °C (Mohamed et al. 2024).

**Table 1:** Primers sequence employed in the experiment

Marker	Forward sequence	Reverse sequence
*OC*	CGCCTGGGTCTCTTCACTAC	CTCACACTCCTCGCCCTATT
*CEMP1*	CCACACCTCAAAATCATCCTGCAGC	ATGGTTGGTCCACAGGGCTAGC
*OPG*	CTAATTCAGAAAGGAAATGC	GCTGAGTGTTCTGGTGGACA
*RUNX2*	GTTATGAAAAACCAAGTAGCCAGGT	GTAATCTGACTCTGTCCTTGTGGAT
*DSPP*	TCACAAGGGAGAAGGGAATG	TGCCATTTGCTGTGATGTTT
*IL-6*	ACTCACCTCTTCAGAACGAATTG	CCATCTTTGGAAGGTTCAGGTTG
*NF-Kβ*	TTACGGGAGATGTGAAGATG	ATGATGGCTAAGTGTAGGAC

*OC*, osteocalcin; *CEMP1*, cementum protein 1; *OPG*, osteoprotegrin; *RUNX2*, run-related transcription factor 2; *DSPP*, dentin sialophosphoroprotein; *IL-6*, interleukin-6; NF-Kβ; *NF-κB*, nuclear factor kappa beta.

### Oxidative stress

The concentration of reduced glutathione (GSH) was measured to determine oxidative stress in cells cultured with both vitamins. Cells were trypsinized, and the cell suspension was centrifuged at 300 × *g* for 5 min. The medium was aspirated, and the cells were subjected to repeated freeze–thaw processes multiple times until complete cell lysis was achieved. The supernatant was collected for further analysis using a commercial kit (#GR 25 11; Bio Diagnostic, Egypt). 5,5′-Dithiobis (2-nitrobenzoic acid) (DNTB) is reduced by glutathione (GSH) within the sample, resulting in the formation of the yellow product, 5-thio-2-nitrobenzoic acid (TNB). The absorbance of the reduced chromogen was measured at 405 nm using a plate reader (BMG Labtech, FLUOstar Omega, Germany) (Tipple & Rogers 2012).

### Statistical analysis

The statistical program SPSS (version 22.0; SPSS, Inc., Chicago, IL, USA) was used to analyze the data. All experiments were conducted at least three times and included three replicates. Quantitative data are presented as mean ± standard deviation (SD). The unpaired *t*-test was used for statistical analysis of the mechanical properties and cytotoxicity data, and the ANOVA test and Tukey post-hoc analysis were used for all other studies. Statistical significance was defined as a *p*-value of less than 0.05.

## Results

### Stem cell isolation and characterization

**Fig. 1:**
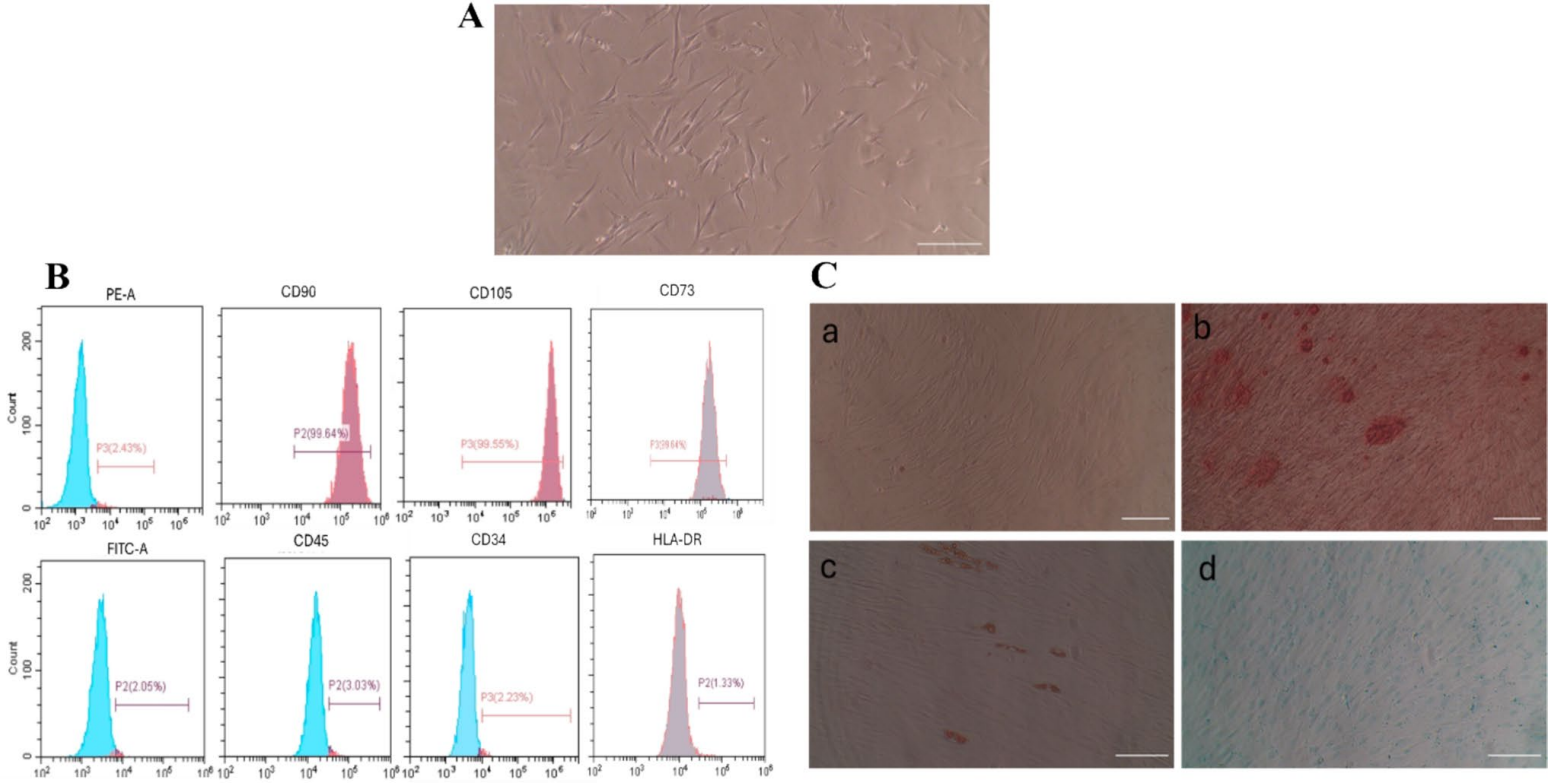
Isolated hPDLSCs characterization. **A** Photomicrograph illustrating the hPDLSCs morphology at P0 (scale bar = 250µm). **B** Flow cytometric analysis of isolated hPDLSCs. The results showed that 99.83%, 98.18%, and 97.59% of the cell populations were positive for CD105, CD90, and CD73 expression, respectively. The cell population showed relatively absent hematopoietic CDs such as CD45, CD34, and HLA-DR.** C** Multilineage differentiation of hPDLSCs after 3 weeks of culture in a specific induction medium (scale bar = 250µm). (a) Undifferentiated cells; (b) mineralized nodules indicating positive osteogenesis; (c) oil droplets indicating adipogenesis; (d) positive, blue-stained proteoglycans indicated chondrogenesis

Microscopic examination of the differentiating cells revealed morphological traits associated with stem cells. These included a distinct plastic adherent and elongated appearance as well as the ability to proliferate and form colonies (Fig. 1a). Flow cytometric analysis showed that hPDLSCs exhibited positive expression of CD73, CD90, and CD105, which are typically associated with mesenchymal stem cells (MSCs). Simultaneously, the results showed a lack of expression of hematopoietic stem cell markers CD34, CD45, and HLA-DR, confirming that these cells possess the properties of MSCs (Fig. 1b).

Following exposure to different culture media (osteogenic, adipogenic, and chondrogenic), the hPDLSCs differentiated into three different lineages. This was confirmed by observations of morphological changes and the use of specific stains (Fig. 1c). Osteogenesis was validated by staining mineralized nodules with Alizarin Red S, whereas adipogenesis was confirmed by staining the resultant oil droplets with Oil Red. Finally, chondrogenesis was confirmed by staining the mucopolysaccharide matrix with Alcain Blue.

### Viability assay results

**Fig. 2:**
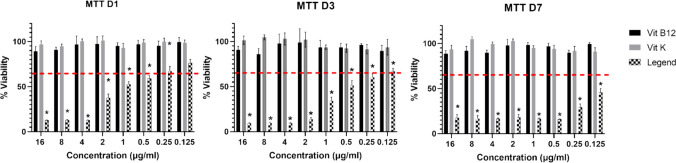
MTT assay results showing mean values of the viability of hPDLSCs cultured with serial dilutions of Vit B12 and Vit K2 (16–0.125 µL) on day 1.3 and 7. Unpaired Student’s *t*-test was performed; *p < 0.05. Results are demonstrated as mean % viability ± SD, the experiment was conducted three times (*n* = 6). The red dotted line represents the threshold for cytotoxicity level (70%) compared to the control

The results of the MTT assay (Fig. 2) showed that neither vitamin had a negative effect on the viability of hPDLSCs. All dilutions were similarly cytocompatible at all observation points (*p* > 0.05), with a viability of more than 70%. Therefore, the highest concentration (16 µg/mL) was used in all subsequent experiments. In contrast, EDTA showed significant cytotoxic effect on stem cells in all determined time points (*p* < 0.0001) compared to both vitamins. Therefore, EDTA group was excluded from the subsequent experiments.

### Scratch wound assay

**Fig. 3:**
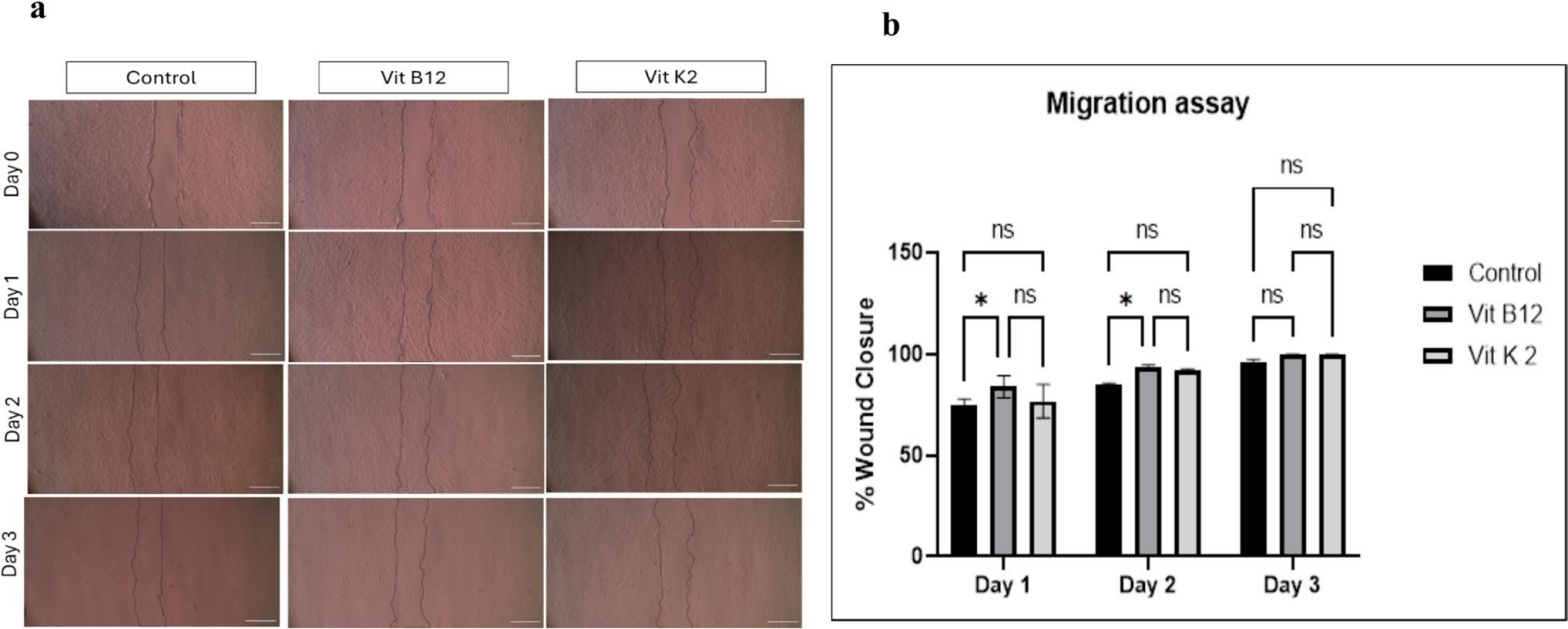
Scratch assay results. Cells were incubated with culture medium conditioned with Vit B12 and K2 for 3 days after scratch induction. **a** Representative images of scratches on day 0, 1.2, and 3 (scale bar = 250 µm). The original wound size is lined with black lines showing that the scratch is imaged from the same place over the designated time intervals. **b** Statistical analysis of the wound area % calculated using ImageJ software. One-way ANOVA and Tukey’s post hoc tests were conducted. Results are demonstrated as mean % wound closure area ± SD, *p < 0.05. The experiment was conducted three times (*n* = 3)

Cell migration was monitored in all groups from day 1 to 3 (Fig. 3a). Statistical analysis (Fig. 3b) showed that there were no significant differences in the migration of hPDLSCs exposed to either vitamin at any observation points (*p* > 0.05). However, the migration of hPDLSCs exposed to both vitamins was significantly faster than that of the control group on days 1 and 2 (*p* < 0.05).

### Scanning electron microscopy

**Fig. 4:**
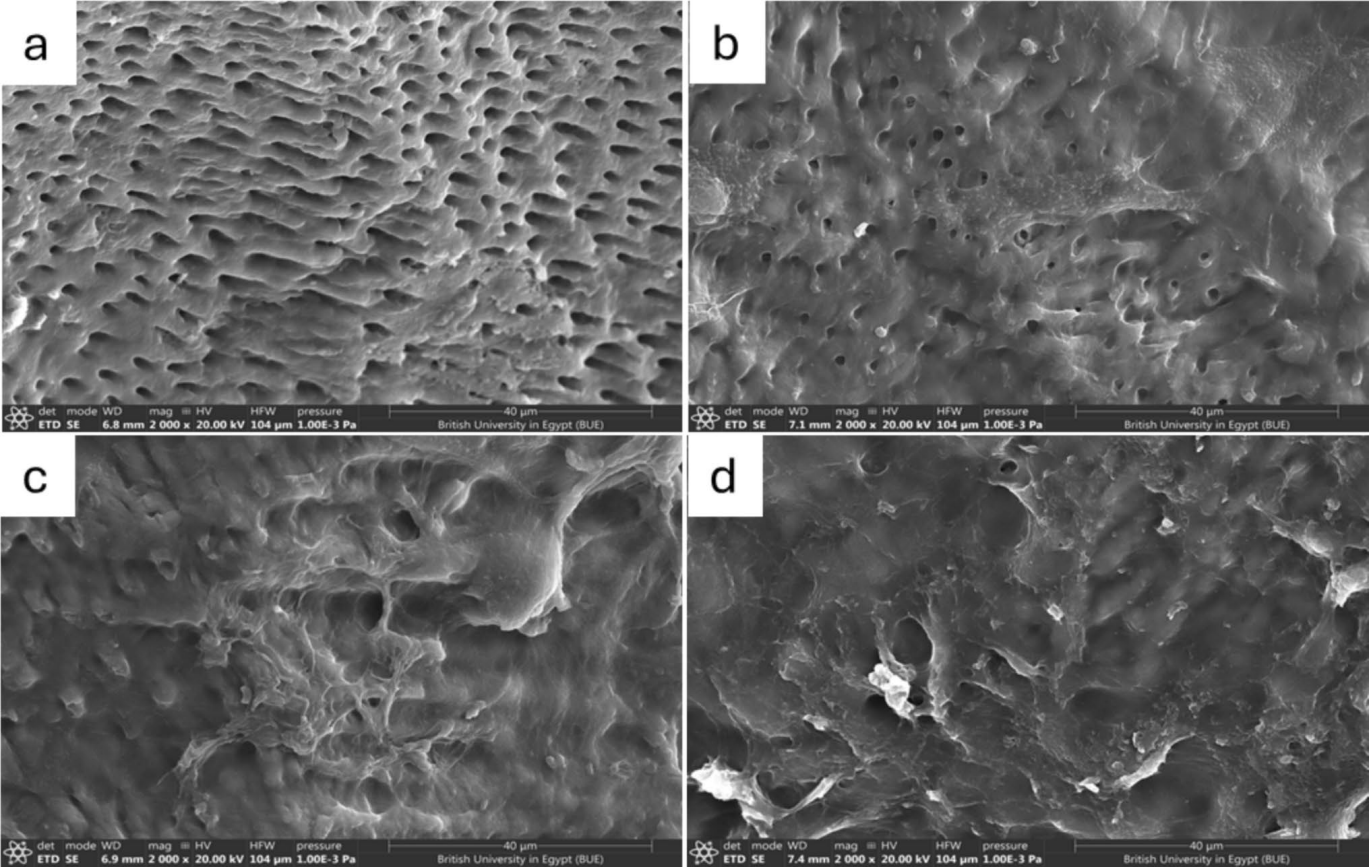
Representative images of hPLDSCs attachment to dentin disks (*n* = 3). **a** Dentin disks without hPLDSCs, **b** hPLDSCs attachment on dentin disks after 48-h incubation, **c** hPLDSCs attachment on dentin disks after 48-h incubation in culture medium supplemented with vit B12, **d** hPLDSCs attachment on dentin disks after 48-h incubation in culture medium supplemented with vit K2

The attachment of hPDLSCs was assessed by imaging the cells on the dentin disks using SEM. Cells showed high attachment to all dentin disks, with characteristic elongated pseudopodia (Fig. 4b, c, d). Layers of stem cells were observed on dentin disks incubated with Vit B12 and Vit K2, denoted by the disappearance of dentinal tubules (Fig. 4c, d) that appeared clearly in normal dentin disks (without cells) (Fig. 4a).

### Alizarin red assay

**Fig. 5:**
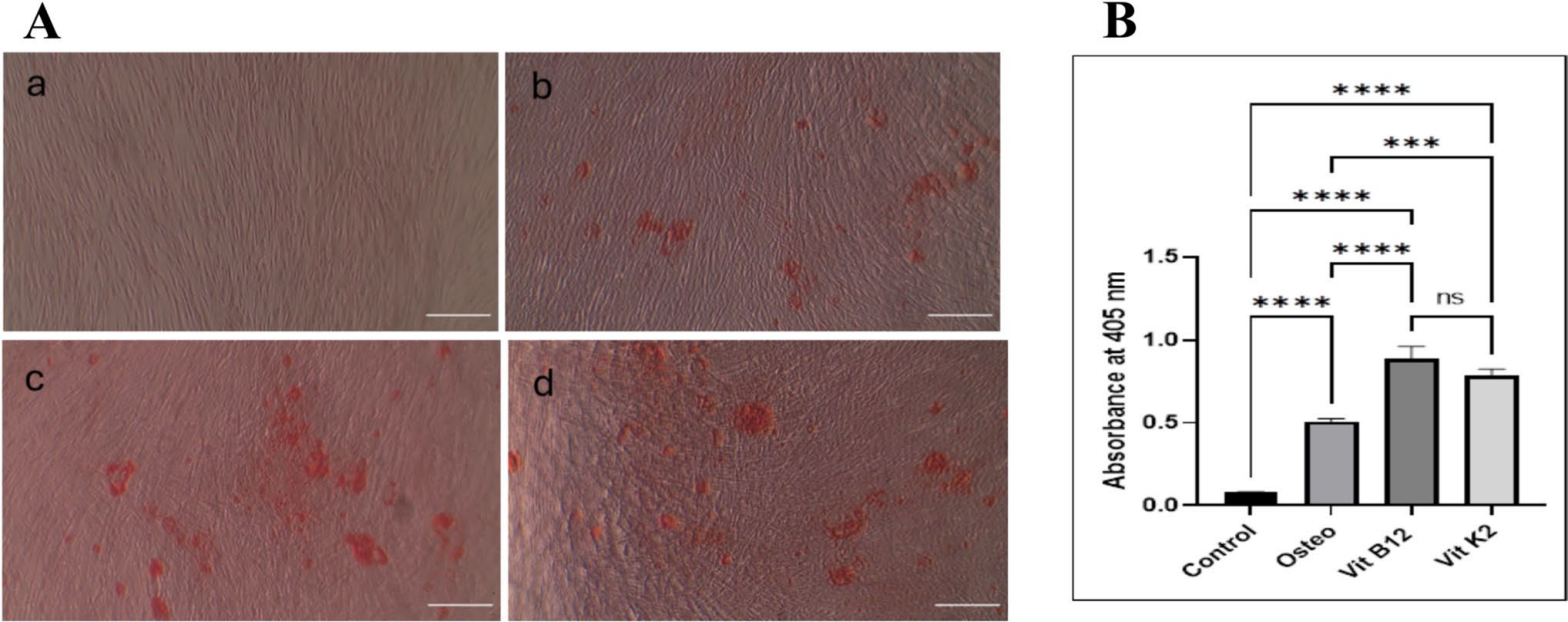
Alizarin Red assay results. **A** Representative images of mineralized nodules after 14 days of hPDLSCs osteogenic induction (scale bar = 250µm). (a) Control undifferentiated group; (b) Osteo group as positive control without vitamins; (c) cells cultured in osteogenic medium supplemented with Vit B12; (d) cells cultured in osteogenic medium supplemented with Vit K2. Data are presented as mean ± SD of 3 experiments. *p < 0.05, **p < 0.01, ***p < 0.001, ****p < 0.001. Statistical analysis employed one-way ANOVA and Tukey post hoc analysis; ns, non-significant

Alizarin red staining on day 14 (Fig. 5) demonstrated that mineralized nodule formation by hPDLSCs cultured with Vit B12 and Vit K2 was significantly higher than that of the positive and negative control groups (*p* < 0.001).

### ALP activity

**Fig. 6:**
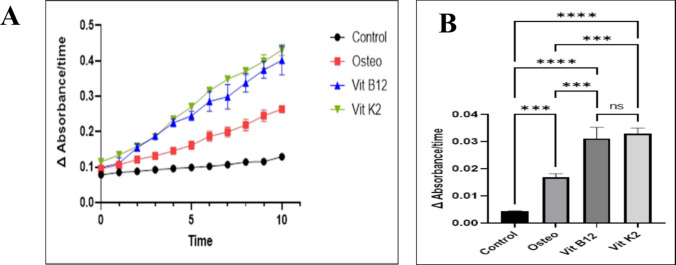
ALP enzyme activity among the study groups after osteogenic induction of hPLDSCs for 14 days. **A** Kinetic profile of ALP enzyme activity by plotting the absorbance of the yellow color product against time. **B** Statistical analysis of the slope of reaction in each group, denoting the rate of ALP activity. One-way ANOVA was performed with Tukey’s post hoc analysis. ***p < 0.001, ****p < 0.001; ns, non-significant

The ALP assay revealed that hPDLSCs cultured with Vit B12 and Vit K2 showed the most rapid increase in p-NP concentration over time (Fig. 6a), indicating increased ALP enzyme activity. Statistical analysis of the results (Fig. 6b) showed that Vit B12 and Vit K2 enhanced ALP activity significantly (*p* < 0.001) more than the positive and negative control groups (*p* < 0.05). However, there was no significant difference between them (*p* > 0.05).

### Osteo/odontogenic and cementogenic markers PCR results

**Fig. 7:**
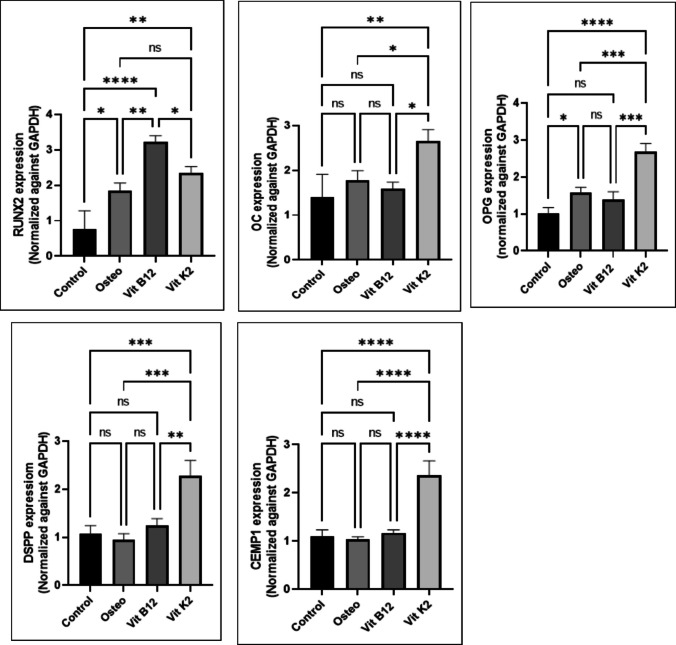
Osteo/odontogenic and cementogenic gene markers expression after 14 days of osteogenic induction of hPLDSCs. The experiment was conducted three times, and each sample was performed in triplicate (*n* = 3). *p < 0.05, **p < 0.01, ***p < 0.001, ****p < 0.001; ns, non-significant. Statistical analysis employed one-way ANOVA and Tukey post hoc analysis

The results showed that after 14 days of osteogenic induction, hPDLSCs cultured with vitamins B12 and K2 possessed different expressions of osteo/odontogenic and cementogenic gene markers compared to the Osteo and control groups. Statistical analysis showed that hPDLSCs cultured with Vit B12 had a significantly higher RUNX2 expression (*p* < 0.05). hPDLSCs cultured with Vit K2 showed significantly higher expression of OPG, OC, DSSP, and CEMP1 (Fig. 7).

### Proinflammatory markers PCR results

**Fig. 8:**
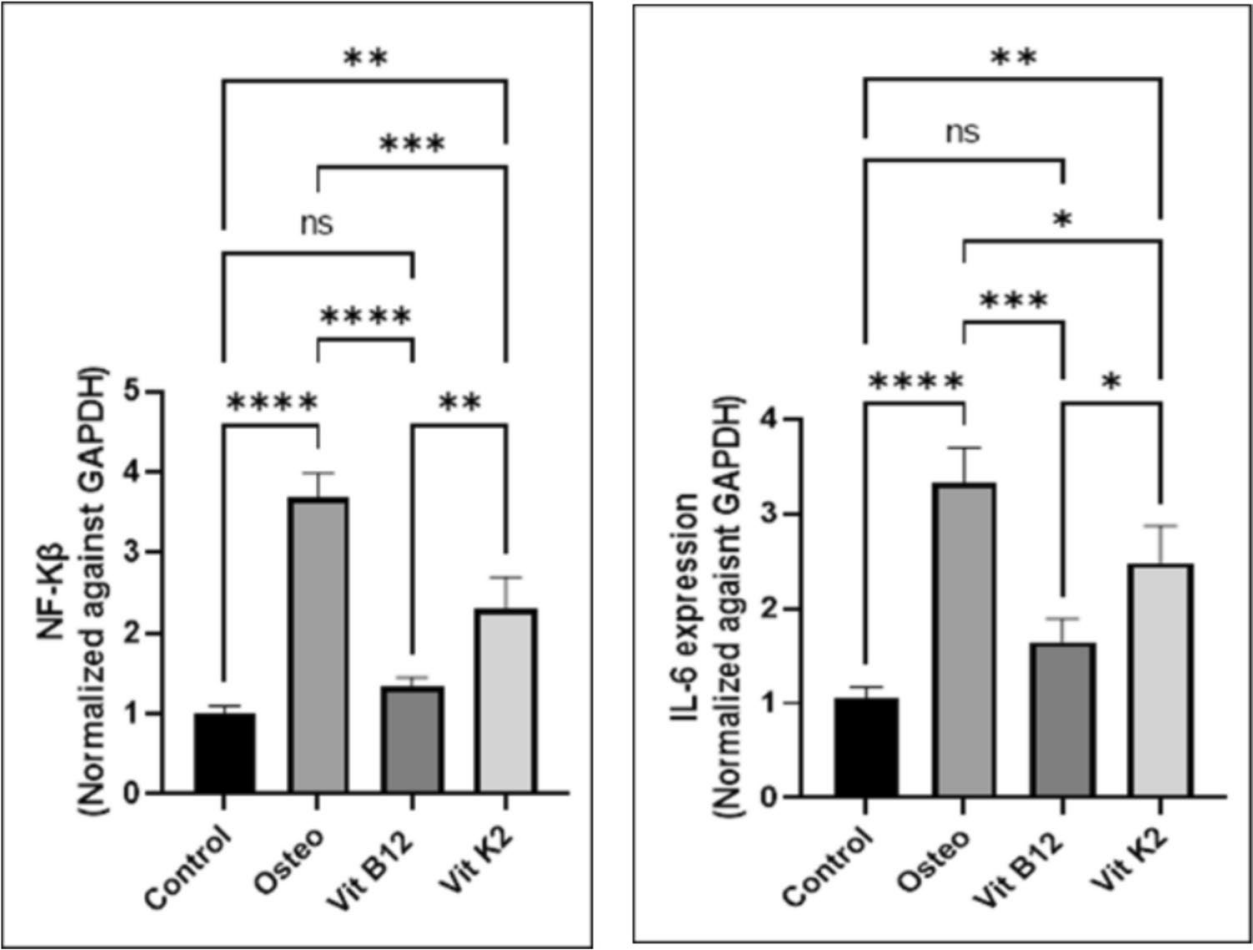
Expression of NF-Kβ and IL-6 pro-inflammatory markers among the study groups after 14 days of hPLDSCs osteogenic differentiation. The experiment was conducted three times, and each sample was performed in triplicate (*n* = 3). *p < 0.05, **p < 0.01, ***p < 0.001, ****p < 0.001; ns, non-significant. Statistical analysis employed one-way ANOVA and Tukey post hoc analysis

Statistical analysis showed that both vitamins decreased pro-inflammatory marker levels compared to the control groups. Vit B12 significantly decreased the expression of NF-Kβ and IL-6 compared to that of Vit K2 (Fig. 8), highlighting that Vit B12 possesses stronger anti-inflammatory properties.

### Oxidative stress

**Fig. 9:**
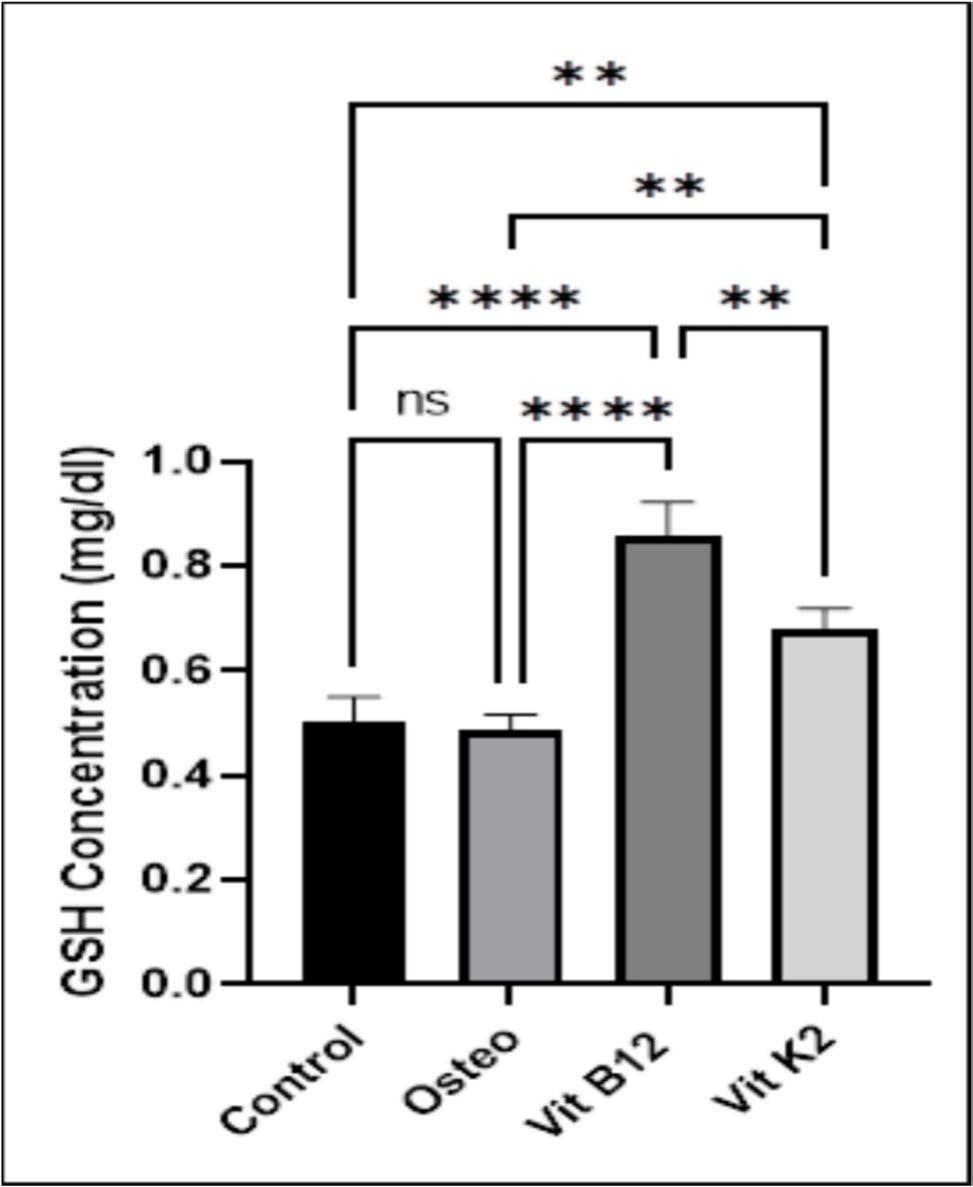
Reduced GSH levels among the study groups. The experiment was conducted three times, and each sample was performed in triplicate (*n* = 3). **p < 0.01, ***p < 0.001, ****p < 0.001; ns, non-significant. Statistical analysis employed one-way ANOVA and Tukey post hoc analysis

Oxidative stress in the study groups was assessed by determining the reduced GSH levels. Statistical analysis showed that cells cultured with Vit B12 and K2 possessed higher GSH expression levels (*p* < 0.01 compared to the Osteo and control groups; however, cells cultured with Vit B12 showed a significant (*p* < 0.01) increase in the GSH level compared to those cultured with Vit K2. These results show that Vit B12 may possess a higher antioxidant capacity than Vit K2 (Fig. 9).

## Discussion

REPs are among the most actively investigated endodontic research modalities. The generally promising results of the biomimetic process, coupled with its multifactorial nature, encourage researchers to explore different avenues of optimization. For example, many developments within the domain of REP have been directed towards enhancing the properties of scaffolding biomaterials, which must be characterized by safety, biodegradability, biocompatibility, and low immunogenicity, in addition to the capacity to support cellular proliferation (Galler et al. 2011). Other studies have evaluated different stem cell sources such as stem cells derived from human exfoliated deciduous teeth (SHEDs), dental pulp stem cells, periodontal ligament stem cells (PDLSCs), and dental follicle precursor cells (Shretha et al. 2016; Alghailan et al 2017; Tsutsui et al. 2020; Sadaghiani et al. 2022). Within this diverse and evolving research landscape, the present study focuses on a complementary strategy: the use of bioactive micronutrients to condition dentin substrates and enhance the regenerative behavior of PDLSCs (Galler et al. 2016b).

The study design targeted generalizability and clinical relevance. In this study, we employed hPDLSCs due to their multipotency, self-renewal capacity, and ability to differentiate into odontoblasts, cementoblasts, and osteoblast-like cells (Park et al. 2011; Lei et al. 2021). More importantly, their use enhanced the clinical relevance of our model, as PDLSCs are among the few stem cell types naturally available in current clinical settings. Unlike SHED, PDLSCs do not require additional donor procedures or transplantation, making them more representative of current in situ regenerative processes. Vit K2 was selected because of growing evidence supporting its individual bioactivities as well as its clinical availability, safety profile, and feasibility for future translation into chairside regenerative protocols. Conversely, the direct influence of Vit B12 on osteogenic differentiation of mesenchymal stem cells (MSCs) remains unclear. While one study reported no significant effect (Vaes et al. 2009), another suggested that the incorporation of B12 into the culture medium enhanced the formation of calcified nodules from dental pulp stem cells (Inamoto et al. 2023).

EDTA was used as control because its use in regenerative procedures is well established (AAE, 2018). In agreement with previous reports, exposure to EDTA led to a marked reduction in hPDLSC viability in our model, supporting existing evidence that, despite its efficacy in dentin demineralization and growth factor release, EDTA can exert cytotoxic effects on periodontal and pulp-derived cells when left in prolonged contact (Phothichailert et al. 2023). This aligns with its clinical use, in which EDTA is applied briefly and subsequently flushed from the canal to minimize undesirable biological responses, as well as previous in vitro studies (Martin et al. 2014; Meeprasert et al. 2023; Phothichailert et al. 2023). The decline in viability observed in the present study rendered further investigation of EDTA in downstream assays—such as migration, attachment, or differentiation—scientifically unsound, and therefore, these analyses were not pursued. In contrast, the absence of cytotoxicity with vitamins K2 and B12 highlights a key translational advantage: their biocompatibility suggests the potential for a more sustained presence on dentin surfaces, which may support prolonged bioactivity without compromising cell survival. This distinction reinforces their promise as alternative or adjunctive conditioners in regenerative endodontic strategies.

To comprehensively assess the effect of dentin conditioning on hPDLSCs, we employed mineralization assays, enzymatic activity measurements, and gene expression profiling (Saber et al. 2023b). Functional assays were used to assess the extent of extracellular matrix mineralization, a hallmark of osteoblastic differentiation. This was visualized using Alizarin Red staining, which detects calcium deposits produced by the differentiated cells. ALP activity was also evaluated because it serves as an early marker of the progression of PDLSCs into the osteogenic lineage (Komori 2019). Gene expression analysis provided further insights into the differentiation status of the cells. RUNX2 plays a pivotal role in the early commitment of mesenchymal stem cells to the osteoblastic lineage, and its expression skyrockets in immature osteoblasts and declines as the cells reach maturity (Komori 2019). In contrast, OC is secreted in large amounts during active bone formation, contributing to energy metabolism and bone remodeling; thus, it is considered a late-stage marker of osteoblastic maturation. Additionally, OPG was found to be a key regulator of the RANK/RANKL/OPG signaling axis (Simonet et al. 1997). In addition, CEMP-1, a cementum-associated protein, was investigated because of its recognized role in biomineralization, facilitating octacalcium phosphate crystal nucleation (Fu et al. 2017; Şelaru et al. 2019). Finally, this study took one further step by investigating proinflammatory markers (IL-6 and NF-*Kβ*) and the levels of reduced GSH. IL-6 is a key pro-inflammatory cytokine that orchestrates immune and inflammatory responses and regulating mesenchymal stem cell proliferation, differentiation, and tissue repair (Assoratgoon et al. 2025; Milewska et al. 2020). Since elevated IL-6 reflects the activation of inflammatory cascades and can alter the regenerative capacity of mesenchymal stem cells, its quantification provides a sensitive indicator of how the tested dentin conditioners can modulate the pro-inflammatory microenvironment and cellular responses (Assoratgoon et al. 2025). GSH is a central antioxidant that helps maintain the intracellular redox balance and protects cells from oxidative stress (Bullon et al. 2025). Since inflammation disrupts this balance by depleting GSH and increasing reactive oxygen species, measuring GSH provides an important marker of how a conditioner influences stem cell resilience and oxidative stress regulation (Bullon et al. 2025; Fan & Simmen 2019).

The results of our assays showed that both K2 and B12 significantly enhanced hPDLSCs behavior across all tested endpoints, including viability, proliferation, migration, trilineage differentiation, and mineralization. Thus, the null hypothesis is rejected. However, no statistically significant differences were observed between the two vitamins in any of the assays, indicating a comparable profile. The comparable enhancement observed with both vitamins may reflect their distinct yet converging modes of action on hPDLSCs. Vit K2 is predominantly involved in bone matrix regulation and mineralization, which could explain the enhanced expression of some osteo/odondogenic and cementogenic markers through the Wnt/β-catenin pathway (Mandatori, et al. 2021). Cui et al. (2021) reported that osteogenic differentiation of PDLSCs is significantly enhanced by Vit K2 in vitro. The gene and protein expression levels of ALP, RUNX2, OCN, and Osterix were increased, and this effect was reversed after the Wnt/β-catenin signaling pathway was blocked.

Vit B12 is known to support cellular metabolism, proliferation, and nucleic acid synthesis (Wheeler et al. 2020; Inamoto et al. 2023). Although its primary mechanisms differ from those of Vit K2, both vitamins contribute to the overall regenerative phenotype by targeting complementary pathways, that is, B12 enhances early cellular readiness, and K2 promotes terminal differentiation and matrix maturation (Cui et al. 2021). This functional complementarity may explain the parity in their effects across all evaluated endpoints despite acting through independent biological axes. However, it is important to note that this study did not explore the potential synergistic interactions between these two vitamins.

As for the pro-inflammatory markers (NF-Kβ, IL-6) and GSH results, in our study, both vitamins reduced the proinflammatory markers and enhanced the intracellular GSH levels relative to the controls. Vit K2 has been previously reported to decrease the expression of NF-κB, resulting in the inhibition of osteoclast activity in experimental animals (Yamaguchi and Weitzmann 2011). Vit K2 anti-inflammatory effect is independent of its well-known role in vitamin K-dependent protein (VKDP) carboxylation and may also involve reduced oxidative stress (Takeuchi et al. 2000).

Our results showed that Vit B12 exerted a significantly greater effect than K2 on pro-inflammatory markers expression, indicating superior anti-inflammatory and antioxidant reinforcement. This indicates that B12 conditioning likely renders cells more resilient to inflammatory and oxidative stress during osteogenic induction. In line with our results, a study by Vallés et al. (2020) reported that the B12 anti-inflammatory effect augmented MSC osteogenesis by elevating ALP activity and mineralization in MSC layers grown under osteogenic conditions. The study also clarified that Vit B12 significantly reduced TNF-α levels released by anti-inflammatory macrophages and modulated the expression of osteogenic markers RUNX2, COL1A1, and ALP. Our results open a new opportunity to investigate whether this anti-inflammatory/redox “boost” is purely beneficial for osteogenesis (which requires some ROS signaling), and whether it depends on dose and timing.

Although in vitro biological assays are simpler, faster, and repeatable, they have limitations, and the results need to be cautiously interpreted to avoid over-reaching conclusions. For example, a study like the one in hand investigated stem cell activity in a carefully controlled environment that lacks the exhausting nature of the periapical region in the presence of bacterial infection. Thus, carefully designed clinical studies are required to confirm or refute our findings. Further, future research should investigate whether combined formulations, varied concentrations, or sequential applications of Vit K2 and Vit B12 could produce additive or synergistic effects on hPDLSCs behavior. Such studies could reveal whether targeted micronutrient combinations offer superior outcomes compared with individual applications.

In conclusion, our study investigated the effects of Vit K2 and B12 on hPDLSC viability, proliferation, attachment, osteo/odontogenic potential, and anti-inflammatory and antioxidant capacities. Although both vitamins had different effects on the expression of osteo/odontogenic, cementogenic, pro-inflammatory, and oxidative stress markers, they eventually produced comparable results on proliferation, degree of mineralization, and ALP activity at the same concentrations.

## Data Availability

Data will be made available on request.
